# Processivity and Coupling in Messenger RNA Transcription

**DOI:** 10.1371/journal.pone.0008845

**Published:** 2010-01-28

**Authors:** Stuart Aitken, Marie-Cécile Robert, Ross D. Alexander, Igor Goryanin, Edouard Bertrand, Jean D. Beggs

**Affiliations:** 1 Centre for Systems Biology, University of Edinburgh, Edinburgh, United Kingdom; 2 Institut de Génétique Moléculaire de Montpellier - CNRS UMR5535, Montpellier, France; 3 Wellcome Trust Centre for Cell Biology, University of Edinburgh, Edinburgh, United Kingdom; University College London, United Kingdom

## Abstract

**Background:**

The complexity of messenger RNA processing is now being uncovered by experimental techniques that are capable of detecting individual copies of mRNA in cells, and by quantitative real-time observations that reveal the kinetics. This processing is commonly modelled by permitting mRNA to be transcribed only when the promoter is in the on state. In this simple on/off model, the many processes involved in active transcription are represented by a single reaction. These processes include elongation, which has a minimum time for completion and processing that is not captured in the model.

**Methodology:**

In this paper, we explore the impact on the mRNA distribution of representing the elongation process in more detail. Consideration of the mechanisms of elongation leads to two alternative models of the coupling between the elongating polymerase and the state of the promoter: Processivity allows polymerases to complete elongation irrespective of the promoter state, whereas coupling requires the promoter to be active to produce a full-length transcript. We demonstrate that these alternatives have a significant impact on the predicted distributions. Models are simulated by the Gillespie algorithm, and the third and fourth moments of the resulting distribution are computed in order to characterise the length of the tail, and sharpness of the peak. By this methodology, we show that the moments provide a concise summary of the distribution, showing statistically-significant differences across much of the feasible parameter range.

**Conclusions:**

We conclude that processivity is not fully consistent with the on/off model unless the probability of successfully completing elongation is low—as has been observed. The results also suggest that some form of coupling between the promoter and a rate-limiting step in transcription may explain the cell's inability to maintain high mRNA levels at low noise—a prediction of the on/off model that has no supporting evidence.

## Introduction

**Figure 1:**
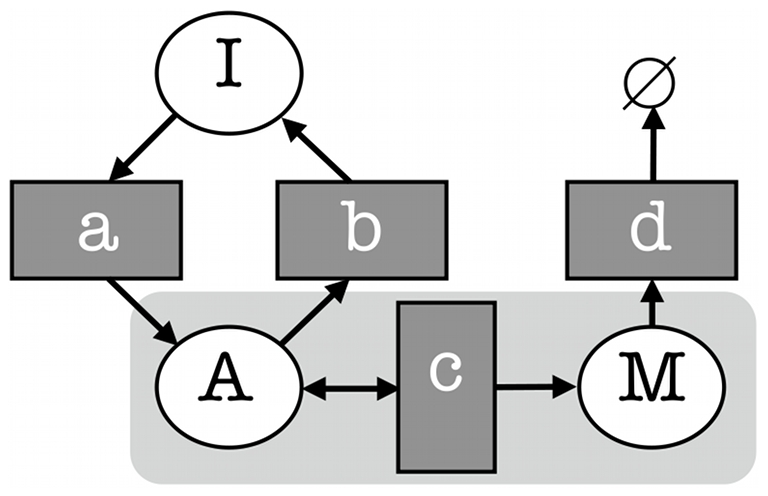
The on/off model of gene activation and synthesis. Circles represent species or states, rectangles represent reactions between species or transitions between states.

The on/off model of gene activation accounts for the noise (standard deviation/mean) observed in mRNA and protein levels in single cells [1]–[7]. This model, shown in Figure 1, explains why cell to cell variations in mRNA may be greater than that of a simple Poisson process [2], [4], that noise strength (variance/mean) varies with the rate of mRNA production [5], and predicts that the same population mean can be achieved with greater or lesser noise as determined by frequency of promoter activation [5]. The variation in mRNA distribution in vivo in yeast has recently been shown to uncover different modes of gene expression, with many yeast genes being less noisy than might be supposed [7]. Noise in protein levels has also been characterised as greater than Poissonian, and has been shown to be pathway specific with stress response genes being among the most noisy [8], [9]. It has also been observed that protein distributions can have longer tails than would be the case if they followed a Poisson distribution, and that this may indicate the existence of generating processes other than a Poisson process [9]. Single molecule studies of *E. coli* proteins have demonstrated that in steady-state they follow a gamma distribution [10], while protein bursts follow a geometric distribution [11]. Noise, or noise strength, is a useful summary of variability, but does not capture any asymmetry in the distribution. Hence, measures of this asymmetry are of interest when analysing models and data.

Imaging experiments are the primary method for obtaining mRNA data at the copy per cell level, and while these are often relatively high-throughput techniques, the resulting histograms often show bin-to-bin variability. When characterising the shape of a distribution, skewness and kurtosis (see Materials and Methods for definitions) provide an alternative to methods that analyse the histogram, such as Kullback-Leibler (KL) divergence. To illustrate the advantage, Table S1 presents the results of a computational experiment where between 100 and 100,000 samples are drawn from a Poisson distribution and the skewness, kurtosis and KL divergence are calculated. This experiment indicates that skewness can be calculated reasonably accurately for sample sizes of 100 and above, while kurtosis may require as many as 1000 samples. For KL divergence, the apparent error between the sampled and the theoretical distribution increases by a factor of approximately 8 for a ten-fold reduction in the number of samples. Therefore, skewness and kurtosis appear to be robust measures with respect to sample size, while KL divergence suffers from the bin-to-bin variability that results from lower sample sizes. We apply skewness and kurtosis to the distribution of mRNA/cell predicted by the on/off model of gene activation. An extensive survey of the parameters of the model is undertaken, where the distributions are computed by stochastic simulation.

The on/off model abstracts from the underlying molecular mechanisms. These lumped processes must be given feasible parameters - a question which we address here by surveying a range of normalised parameters. More substantial questions arise when this phenomenological model is refined into a more detailed model. A more detailed representation of the molecular events in the initiation of transcription is proposed in [12] where two inactive and two repressed states are modelled, in addition to the active state. In more recent work [13], this five state model of the promoter is simplified for analysis using the on/off model. (Note that transcription remains a single reaction in these models.) The many RNA processing events that can occur co-transcriptionally (such as capping, pre-mRNA splicing and 3′ end cleavage and polyadenylation [14]), the coupling between transcription and mRNA export, and the possible interrelationship between the promoter state and transcript elongation are all candidates for inclusion in a more detailed model of transcription. Such a model might account for every nucleotide addition step that the RNA polymerase, Pol II, performs after initiation (for a review of the role of Pol II in transcription see [15], [16], for a discussion of elongation see [17], and for an overview of coupling see [18]). This is important to allow key downstream processing events to be modelled, such as transcription of intron sequences (the 5′ and 3′ splice sites and branchsite). The change in representation required has the effect of making transcription more deterministic - a property we investigate further by stochastic simulation. Step-wise models of elongation are proposed in [19], [20] where it is shown that the elongation time is narrowly-distributed around the mean (for genes of 1kb and where stepping forwards is more likely than backtracking). Pausing is shown to skew the distribution of elongation times positively, that is, increasing the probability of longer elongation times. In the context of translation, a queueing model [21], [22] has been proposed to represent the progress of ribosomes along the mRNA. This model is shown to classify mRNAs on the basis of properties of the flow of ribosomes, in agreement with experiment. The size and duration of a burst of protein or mRNA copied from a biopolymer template are defined in [23] in a model that can be solved for the waiting time between bursts of production.

The elongation models analysed here explore the possibility that a polymerase will continue elongating even if the promoter becomes inactive and, as an alternative, that the gene must be in the active state for elongation to complete successfully. The ability of Pol II to travel the length of the gene is termed processivity in [24], and it was demonstrated that this ability can be uncoupled from the elongation rate. That is, factors that affect processivity do not affect the rate of elongation, but elongation defects reduce processivity through the dissociation of Pol II at sites of arrest. We show that these properties have a significant impact on the distribution of mRNA in terms of skewness and kurtosis, in addition to altering the mean and variance. These changes to the basic model are based on recent observations of the mRNA transcription process made at the single gene level [7], [25]–[27] and form the basis of a more detailed model of transcription being developed to explain elongation and RNA processing in time-series and distribution data. On the methodological level, the simulation results show that when comparing models, skewness differs from model to model by several standard deviations across much of the parameter range, and so may be a valuable addition to the methods used for analysing single-cell data.

This paper continues with a description of the on/off model, discussion of its parameters, and presentation of the simulation results. Next, we explain the elongation models and present the simulation results for them. The investigation of the alternative models concludes with an analysis of mRNA data from yeast, and a survey of published model parameters. Finally, we discuss related work and draw conclusions from our study in this wider context.

## Results

### Stochastic Models and Methodology

The on/off model of transcription is represented as a Dizzy model and simulated using the Gillespie algorithm (see Materials and Methods). Figure 1 depicts the model, and a listing of it can be found in Appendix S1. This model specifies that mRNA (*M*) is synthesised at a rate *c* only when the gene is in the active state, and that mRNA is degraded at a rate *d* in any state where *M* 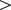
0. The transitions of activation and inactivation have rates *a* and *b* respectively, and move the gene from the inactive (I) to the active (A) state (off to on) and vice-versa.

A useful distinction can drawn between the rate *c* of mRNA production when the gene is in the active state, and the process of transcription as a whole, which is the observable rate of production of mRNA over time, or in a population of cells, and incorporates the modulation of *c* by the on/off switching rates *a* and *b*. We refer to the former as *synthesis* in the following discussion.

An analytic solution exists for the on/off model [28] and will serve as a reference for the simulation-based approach adopted here. Stochastic simulation is an alternative to solving complex sets of equations, and yields the probability distributions for species of molecules, in addition to the mean and variance that can be derived analytically. Computational simulation can also be used to determine the time course of a stochastic model.

### The On/Off Model of Transcription

The on/off model was originally expressed by the following equations:
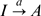
(1)

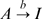
(2)

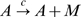
(3)

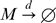
(4)following [28], we analyse the model for *d* 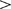
0 (*M* is Poissonian for *d* = 0). (This model was originally named the IAP Process and described the production of protein rather than mRNA.) The expressions for the steady-state mean and variance are as follows, from which the noise strength can be derived:
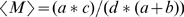
(5)

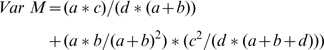
(6)

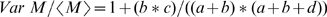
(7)


The steady-state variance has two terms: The first corresponds to the mean, and therefore the ratio of the variance to the mean will always be greater than 1. That is, the noise strength will be greater than that of a Poisson process which has a ratio of 1. It is noted in [4], [28] that the model is normalised by the degradation rate *d*. That is, the degradation rate sets the timescale over which the model evolves to steady state - the characteristics of the model are not otherwise dependent on *d*. This feature is exploited in the following exploration of the model parameters.

### A Survey of Parameters for the Normalised Model

Knowing that the model parameters can be rescaled to plausible values, the behaviour of the model is explored for a normalised degradation rate *d* = 1. It is helpful to structure the analysis by calculating *b* 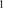 to give a desired value of *M* for selected values of *a* 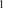 and *c* 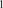, where the subscript 1 denotes normalised parameters. We choose *M* = 1, 2, 5, 10 and record characteristics of the distribution for the parameter combinations that yield the selected mean mRNA level. The survey is limited to low values of *M* as many constitutively expressed yeast genes have mRNA abundances in this range. The statistics of interest are the mean, variance, skewness, kurtosis, noise strength and the histogram of the simulated distribution of *M*. To condense the presentation of the results, we focus on mean values of *M* = 1 and 10.

The values of *a* 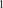 we survey range from 1/8 to 128, and the values of *c* 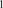 range from 10 to 5000. (The parameter values specify the reaction probability density per unit time.) These ranges are selected to include values determined by [4] to characterise noise in mammalian mRNA (i.e. *a* 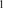 around 1 and *c* 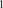 between 500 and 1000). Values of *c* 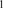 as low as 10 are explored because values of 500 and above may not give plausible synthesis rates on rescaling (this issue is discussed below). Higher values of *a* 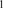 are explored in order to discover the behaviour of a system that transitions more rapidly from off to on, with respect to the degradation rate. Often, 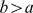 and in most cases 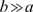. This means that the time spent in the inactive state (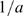) is typically much greater than the time spent in the active state (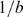). The ratio 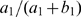 is also of interest as it represents the fraction of time spent in the on state. This ratio is in fact constant for each series of values of *c* 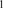 explored here, as *M* is held constant, and by equation 5: 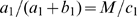. We show that varying *a* 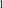 while keeping *M* and 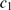 (and therefore this ratio) constant results in a wide range of behaviour.

The analysis presented here is organised around *a* 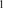. When *a* 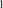 = 1 the time in the inactive state equals that taken for a molecule of *M* to degrade, on average. It should be noted that the two modes of gene expression 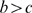 and 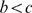 identified in [7] correspond to *a* 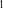 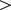
1 and *a* 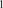 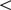
1 for *M* = 1, and to *a* 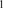 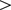
10 *a* 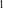 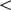
10 for *M* = 10 in the analysis presented here. Modes of expression are discussed in more detail later, where we also show that the range of *a* 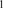 values surveyed includes all those we could obtain from the literature. As we are primarily concerned with noise in the transcription process itself (intrinsic noise), noise strength is taken as the most appropriate measure of variability. It should be noted that noise strength scales with the mean, which can lead to unintended artifacts where both intrinsic and extrinsic noise is considered [29].

### Simulation Results

**Figure 2:**
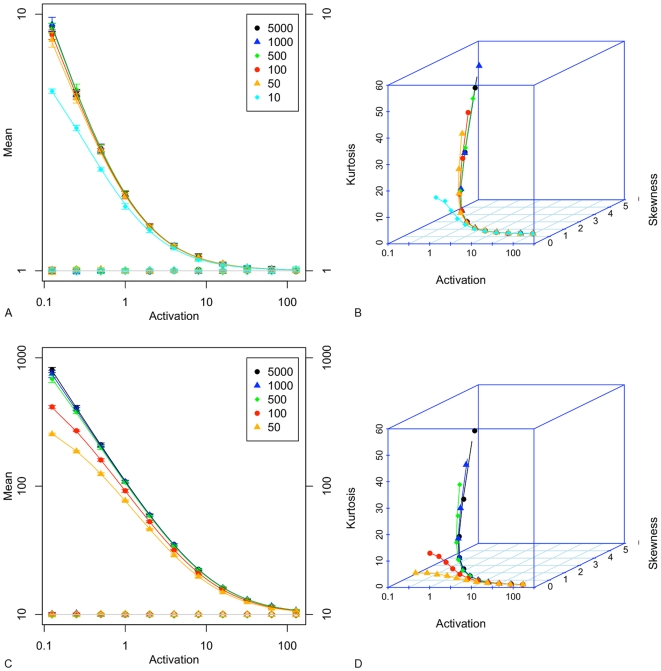
Simulation results for 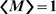. A. mean (open symbols) and variance (filled symbols); B. skewness and kurtosis. For 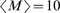: C. mean and variance; D. skewness and kurtosis. Points are average values from 10 repetitions for a given activation 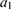 rate, and are colour coded according to 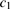. In A. and C., a log 10 scale is used on both axes, and error bars show the standard deviation for 10 repetitions. Solid lines in A. and C. are the theoretical solutions for mean and variance are derived from equations 5 and 6. Solid lines in B. and D. are computed from equation 1 in [4] (Supplementary Material).

Figure 2 (A and C) shows that the simulated values for mean (plotted as open symbols) and variance (plotted as filled symbols) lie on the curves predicted by equations 5 and 6, as would be expected. Note that the color coding used in this plot, and those following, identifies a series of results according to the value of synthesis *c* 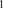, e.g. orange triangles denote models where *c* 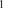 = 50, the independent variable (activation) is on the x axis and the dependent variables on the y axis. Variance is proportionately higher when *M* = 10 in comparison with *M* = 1. In both cases, for activation rates *a* 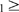
10, the mean and variance tend towards the same value, and hence noise strength tends to 1. Figure 2 (B and D) shows that skewness and kurtosis reduce as *a* 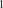 increases from its lowest value, and that the range of skewness and kurtosis values is not strongly dependent on the mean value of *M*. The variation of skewness and kurtosis with *a* 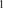 is highest for the largest synthesis rates, *c* 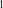, and this reduces considerably for low synthesis rates. Skewness and kurtosis reduce to approximately 1 for a mean *M* = 1 (2B), while for *M* = 10 (2D) they reduce to 0.34 and 0.13 respectively. These results confirm that the second, third and fourth moments of the mRNA distribution are only weakly dependent on *c* 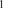 when *a* 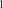 is above 2. The error bars for skewness and kurtosis are plotted in Figure S1.

**Figure 3:**
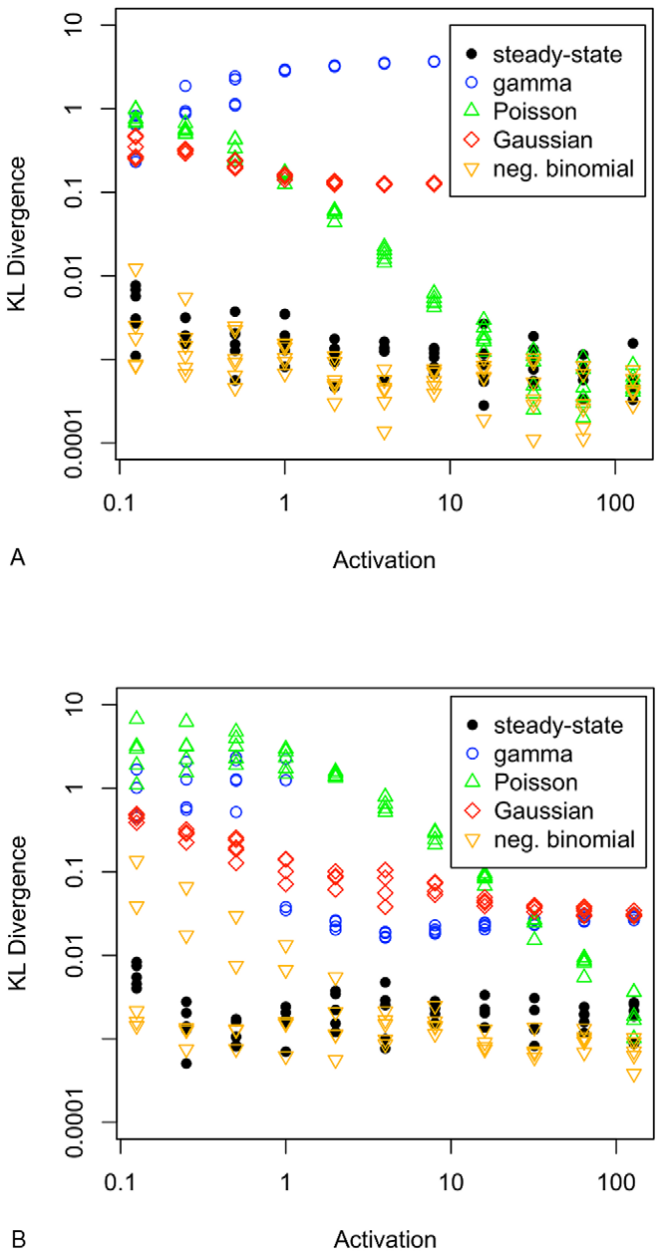
Kullback-Leibler divergence between the simulated distribution at 50s simulated time and that at 33s (the steady-state series), and the best fitting gamma, Poisson, Gaussian and negative binomial distributions. A. 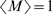, and B. 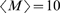. Points are values from a single survey of 66 parameter value combinations (55 in the case 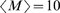), ensuring that each of the standard distributions is fitted to the same simulated distribution and so the KL values are directly comparable.

Figure 3 shows the extent to which the on/off model can be fitted by gamma, Poisson, Gaussian and negative binomial distributions. Neither the gamma nor Gaussian model fits as well as the negative binomial distribution. The Poisson distribution fits well for certain values of activation, depending on the mean *M*. With the exception of *c* 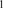 values of 100 and below (*M* = 10), and consistent with the analysis of [30], the on/off distribution is accurately modelled by the negative binomial distribution.

These results suggest the mRNA distribution can never be Gaussian for low mean values, while it can take a more symmetric form as *M* and *a* 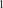 increase. The results account for both the behaviour shown in [3] where the parameters give a Poisson or normal-looking distribution, and that described in [4] where a much noisier parameterization is chosen. A consequence of these findings is that fitting parameters in the space where *a* 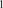 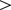
8 will be hampered by the lack of variation of the distribution with *c* 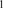. The error bars on skewness and kurtosis show that we could not confidently determine activation rates from these values, although they do vary with *a* 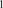.

### Parameter Interpretation

As noted, the degradation parameter *d* specifies the timescale. Letting 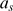, 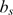 and 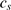 be the parameters for some other time scale 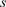 as determined by the degradation rate 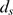, then the scaled model is equivalent to the normalised model providing:
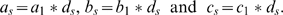



That is, the predictions for the normalised parameters can be rescaled to determine the absolute rates (or absolute mean time for a transition) given knowledge of 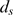. The plausibility of the off-on activation rate can then be assessed, as can the feasibility of the synthesis rate 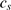. The scaled values for *a* for degradation rates of 1s, 60s, 10min, 1 hour and 4 hours are given in Table S2. The scaled *c* values are given in Table S3.

From considerations of the initiation rate (0.2), physical size (100 bp) and average elongation rate of Pol II (20–30 bp/s), it can be concluded that an mRNA transcript can be completed at a maximum rate of one every 5 seconds, i.e. a rate of 0.2 [31] (assuming also that events in elongation, splicing and termination apply uniformly to transcripts). Allowing for the possibility of a higher maximum elongation rate of up to 70 bp/s [26], we would limit the maximum mRNA production rate 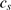 to lie in the range 0.2–0.5. Although inexact, such an estimate is useful as it places constraints on plausible 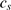 values for a known 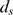. Table S3 lists the scaled values (i.e. the product of the normalised *c* 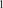 rates and the actual degradation rate). Degradation rates observed for some yeast mRNAs are in the order of minutes while in mammalian systems the rate may be in the order of hours. Taking the half life of a yeast mRNA as 10 minutes, values of 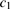 of 500 are not plausible as they correspond to mRNA being transcribed above the maximum rate. This value for *c* 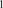 is plausible for mammalian mRNA synthesis where the degradation rates can be 4 hours [4]. By considering how the normalised parameters are scaled, we can constrain the plausible range for model parameters. Naturally, our beliefs about plausible rates may change as new evidence emerges.

Once the parameters have been rescaled, the average times a gene spends in the active and inactive states can be worked out - we can consider whether it is plausible for a gene be active for 1/10s, or for 100s. This requires biological insight and a deeper model of transcription that incorporates events at the molecular level.

### Models of Elongation

The production of a mature eukaryotic messenger RNA involves many processing events that can occur co-transcriptionally, including 5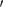 end capping, intron removal (splicing) and 3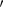 end cleavage and polyadenylation [14]. Some of these events can now be observed and measured at the single-transcript level. The extent to which these transcription and processing events are coupled is still under investigation and stochastic modelling may play a role in formulating and testing theories. However, the on/off model clearly does not account for the many hundreds of molecular events that occur in the production of a transcript, nor of the abortive transcriptional activity that is now being uncovered. In this section, we examine several modifications that change the characteristic of the synthesis step by representing the elongation process in more detail. It should be noted that elongation is not typically considered the rate limiting step in synthesis, the prior initiation step will set the rate for the synthesis of mature transcripts. However, under high induction the spacing of Pol II sets an upper limit as discussed above. And if, in addition, the gene is short, we can consider the case where elongation does become the sole and limiting factor.

**Figure 4:**
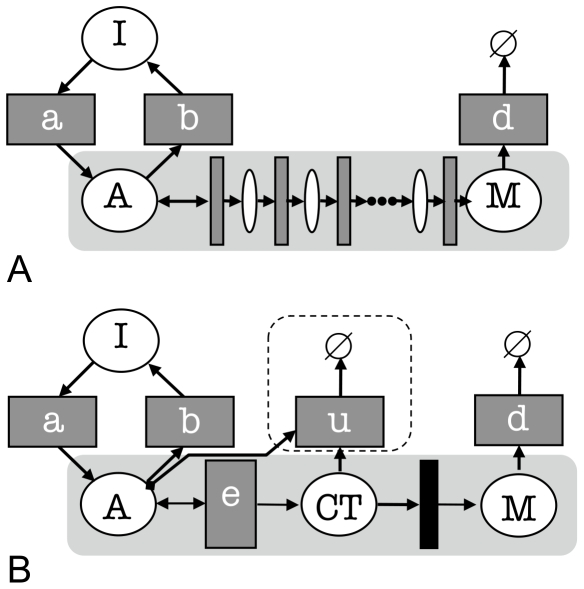
Modelling initiation, synthesis and the coupling between them. A. synthesis is modelled by a series of steps; and B. by a counting procedure, where (optionally) the count may decrement. The filled rectangle represents a transition that is enabled when a threshold is reached, i.e. when the count reaches N.

A model of elongation might represent every base (or nucleotide) addition event for each of the N bases in the transcript. Figure 4A shows how the elongation process might be incorporated into the on/off model. Rather than fix a value for N to model a specific gene, we note that, for any large N, synthesis is now modelled as a more deterministic process [19], [25]. A parameterization for the model is obtained by giving each of the N transitions a rate N times that of the original synthesis step of the on/off model. The key questions are how the mRNA distribution is affected by modifying the model in this way, whether these changes are reflected in observable differences in the mean, variance, skewness and kurtosis, and whether any such changes can be used in model fitting and model selection.

In exploring the parameter space, we make the simplifying assumption that at most one Pol II is active at any time, and hence ignore any effects of polymerase assemblies holding each other up during elongation (as noted earlier, this phenomena has been investigated by [19], [20], [23]). This assumption is borne out for some yeast genes where typically 0 or 1 nascent mRNAs are detected, for example MDN1 [7], (while an average of two polymerases were observed at the transcription site in [26]), but clearly this assumption will not apply to longer genes expressed at high levels where multiple Pol IIs transcribe mRNA simultaneously. If the gene is short, and the induction level is high, then we may ignore the time between the completion of each round of transcription and effectively consider elongation as the rate setting step. Were Pol II to be recycled at the promoter, we would have exactly this case. This simplification mirrors that in the original on/off model where the initiation rate and the time for synthesis cannot be distinguished from the synthesis rate.

In the processive and coupled elongation models, the steps in elongation are represented by a counting procedure rather than by explicitly creating a series of N transitions. This is preferable as simulating models containing long series of transitions is more computationally expensive than the counting approach, and the models they implement are equivalent. The elongation transition increments a count place *CT*. When the count reaches a specified value the number of mRNA *M* is increased by 1 (as indicated by the filled rectangle that represents the testing of the count value, see Figure 4B). Letting N = 100, the synthesis rate is multiplied by 100 to obtain the elongation rate *e*. When the gene is active, the elongation process takes the same amount of time as the original synthesis step, and so the rate of mRNA production should be the same as in the original model. But the variability of the synthesis time is much reduced, and so the effect on the distribution characteristics must be investigated. The elongation process is modelled stochastically, but has a more predictable outcome. A narrow distribution of elongation times also follows from similar assumptions made in [19]. As the elongation rate is easily derived from the *c* 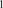 parameter, it is straightforward to assess the behaviour of the new model using the same *a* 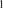 and *b* 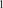 parameters as before. No analytic solutions are available for the models described below; however, it is possible to show the effects of the modifications by comparison with simulations of the on/off model (or the analytic solutions for it).

### Processive Elongation - the On/Off-PE Model

In a simple deterministic elongation model, consistency with the on/off model would be retained as all elongation events contribute to *M* (see the Text 1 for more details of this model). However, when the count is interpreted as corresponding to the position of a Pol II on the gene, it is implausible that the count (Pol II location) is retained from one round of activation to the next. In fact, elongation is often considered to be processive, that is, Pol II assemblies will continue to transcribe mRNA even if the promoter becomes inactive. The on/off-PE model allows the count, once started, to continue until *M* is incremented once, irrespective of the gene being active or inactive. This modification may allow an additional mRNA to be produced on each round of transcription. And, as the limiting reaction rate is greater than in the on/off model, this effect will be magnified at higher activation rates. (This magnification would be less were initiation an explicit step in the process: The processive model represents the upper limit for an initial rate-limiting step in synthesis.)

### Coupled Elongation - the On/Off-CE Model

Another possibility is for Pol IIs that are part-way through elongation when the gene transitions to the inactive state to dissociate from the DNA. (The inefficiency of engagement and elongation has been described in [26].) The on/off-CE model modifies the deterministic elongation on/off model by resetting the count when the gene becomes inactive. This change removes the contribution of previous elongation activity when the gene re-activates, and implements a simple coupling between the completion of elongation (which we equate with the production of a mRNA) and the state of the promoter. Elongation can be ‘abortive’ - elongation may not lead to a mRNA - and its successful completion is correlated directly, or indirectly, with the promoter.

### Simulation Results

**Figure 5:**
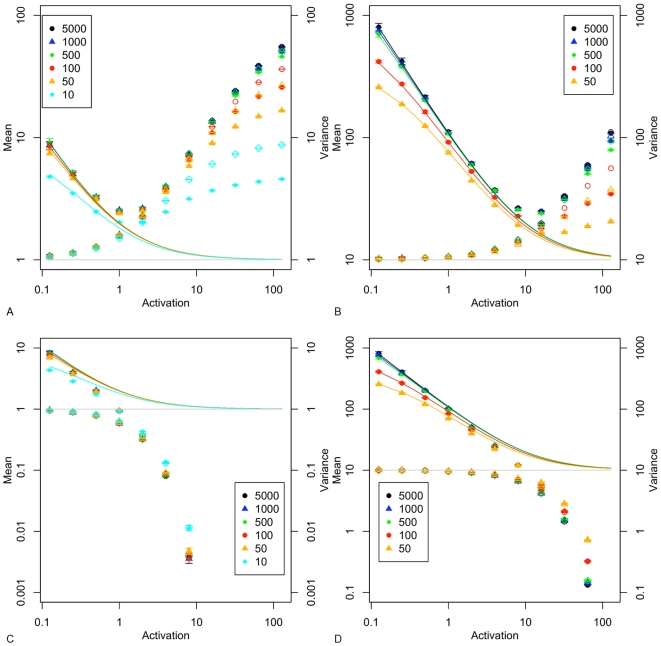
Mean (open symbols) and variance (filled symbols) for the on/off-PE model. A. 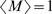; B. 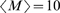. For the on/off-CE model: C. 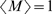; D. 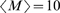. Points are average values from 10 repetitions. Error bars show the standard deviation for 10 repetitions. Solid lines are the theoretical solutions for mean and variance.

Beginning with the results for mean and variance for the on/off-PE model, Figure 5A shows that for a desired *M* = 1, the simulated mean rises above the desired value for all but the lowest values of *a* 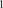. For *a* 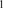 = 2, the mean is approximately double that intended. The mean continues to rise as *a* 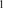 increases, the range of values for the steady-state mean lies in the range 1.05–55.5 copies/cell. The variance takes the predicted values for *a* 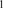 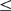
1, but thereafter increases in parallel with the increase in the mean. The noise strength does not decrease below 0.9 except for the combination of *a* 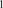 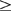
4 and *c* 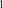 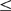
100. When *M* = 10 (Figure 5B), the mean values for *M* predicted by the on/off-PE model begin to rise above the desired value of 10 as *a* 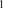 increases beyond 8. Mean mRNA lies between 10.1 and 111 copies/cell across the parameters surveyed. However, noise strength only reduces significantly below 1 for the combination of high values of *a* 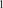 and low values of *c* 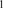, as the increase in the mean is again matched by increasing variance.

Figure 5 (C and D) shows that for the on/off-CE model when *M* = 1, mean and variance both fall significantly below their predicted values as *a* 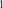 increases above 2. This is due to the increasing probability that the gene will switch to the off state while elongation is in progress, a consequence of which is the reduced overall rate of mRNA production. Noise strength tends to 1 as *a* 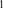 increases. Despite mean and variance diverging from the values predicted by the on/off model, they converge, and so the noise strength characteristic does not appear different from that of the on/off model.

**Figure 6:**
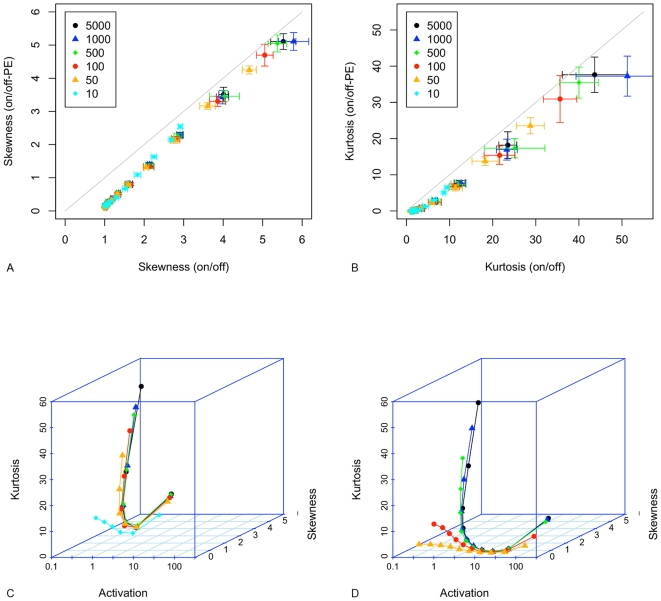
Scatterplots of skewness and kurtosis taking the on/off model as a reference (plotted on the x axis). A. skewness for the on/off-PE model. B. kurtosis for the on/off-PE model. 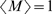 in A and B. Skewness and kurtosis for the on/off-CE model for C: 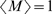; and D. 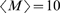. Points are average values from 10 repetitions. Error bars, where shown, indicate the standard deviation for 10 repetitions. Solid lines join points in the same series.

The skewness and kurtosis of the on/off-PE model are compared to those of the on/off model in the scatterplot in Figure 6 A and B for *M* = 1 (and in Figure S2 for *M* = 10). The on/off-PE model has correlated, but reduced, skewness and kurtosis (with reference to the on/off model). As can be seen, the predictions differ by 2 or 3 standard deviations over much of the parameter space. The on/off-CE model shows a different characteristic, as indicated in Figure 6 C and D, where both measures vary non-monotonically, reducing as *a* 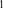 increases from 1/8 to 2, then increasing again as the *M* distribution becomes increasingly concentrated around 0. For the on/off-CE model, when *M* = 1, skewness has a minimum value of approximately 2, and kurtosis remains above 4; and when *M* = 10, skewness has a minimum value of 0.54, and kurtosis remains above 0.34.

In summary, the models considered here have very similar characteristics when *a* 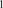 is less than 2, and *M* is high (i.e. 10). It is most evident that different mechanisms are in play for low values of *M*. Differences are also observed when *a* 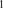 is greater than 2: It has been shown that using an elongation model as the representation of synthesis in the on/off model can explain values of noise strength below 1. The range of skewness and kurtosis values is shown to be largely independent of the mean, hence re-scaling is not necessary in order to compare values across datasets or models (i.e. in contrast with the variance, factoring by the mean is not necessary). Skewness values show differences of several standard deviations when comparing the deterministic, -PE and -CE models with the reference model, and this may be a general phenomena that is useful in model fitting and model selection.

The mechanisms proposed in the elongation models can be reviewed in the light of the simulation results. For more frequent activation/inactivation cycles, the processivity mechanism increases mRNA production significantly. The high probability of elongation initiating, with no means for elongation to pause or for Pol IIs dissociate, is the reason for this outcome. Were the probability of the (single) Pol II completing elongation to be 1/90, as observed in [26], then the mean mRNA would be much reduced. A reduction of this scale cannot be applied when *a* 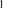 is low without stopping elongation, but could be a factor that reconciles the processive model with the basic on/off model for high values of *a* 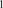. In contrast, the coupling mechanism causes mRNA levels to be depressed. This suggests that for higher activation rates (*a* 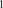 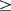
8), any coupling between the promoter and a rate limiting step in synthesis will prevent high mean mRNA levels being achieved at low noise. This effect is observed for the particular case where elongation is rate limiting, but would also follow if the escape of Pol II from the promoter-proximal pause region was linked to promoter state.

### Modelling the Single-Cell

Two questions are now addressed in the analysis of single-cell data: i. do skewness and kurtosis measured from real data match values predicted from the model? and ii. is there evidence of constraints on the parameters of the on/off model when the model is fitted to single cell data?

**Figure 7:**
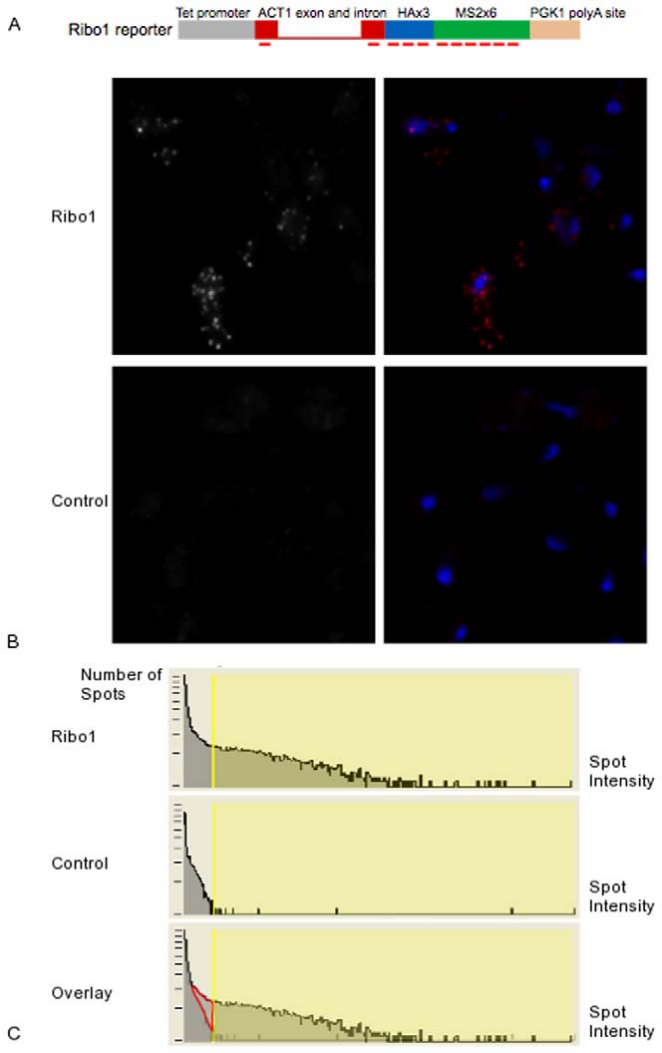
Image-based measurement of mRNA in yeast. A. Schematic of the Ribo1 reporter gene used in this study. The position of the probes used to detect the reporter RNA are indicated by horizontal red bars. B. Detection of single molecules of the Ribo1 reporter RNA. Expressing (upper panels) and control cells (lower panels) are shown. Left panels display the RNA signal (red) overlayed with the nuclei (blue). Each field in a projection of a 3D stack (18×18×6 µm). C. Efficiency of RNA detection. Histograms of the number of spots versus spot intensities across an entire 3D stack (63×63×6 µm) are shown for control or Ribo1 expressing cells. The area shaded in yellow correspond to the spots included in the analysis after thresholding. Bottom panels: overlay of the two histograms revealing the amount of Ribo1 RNA molecules lost by the thresholding procedure (<15%; red area).

**Figure 8:**
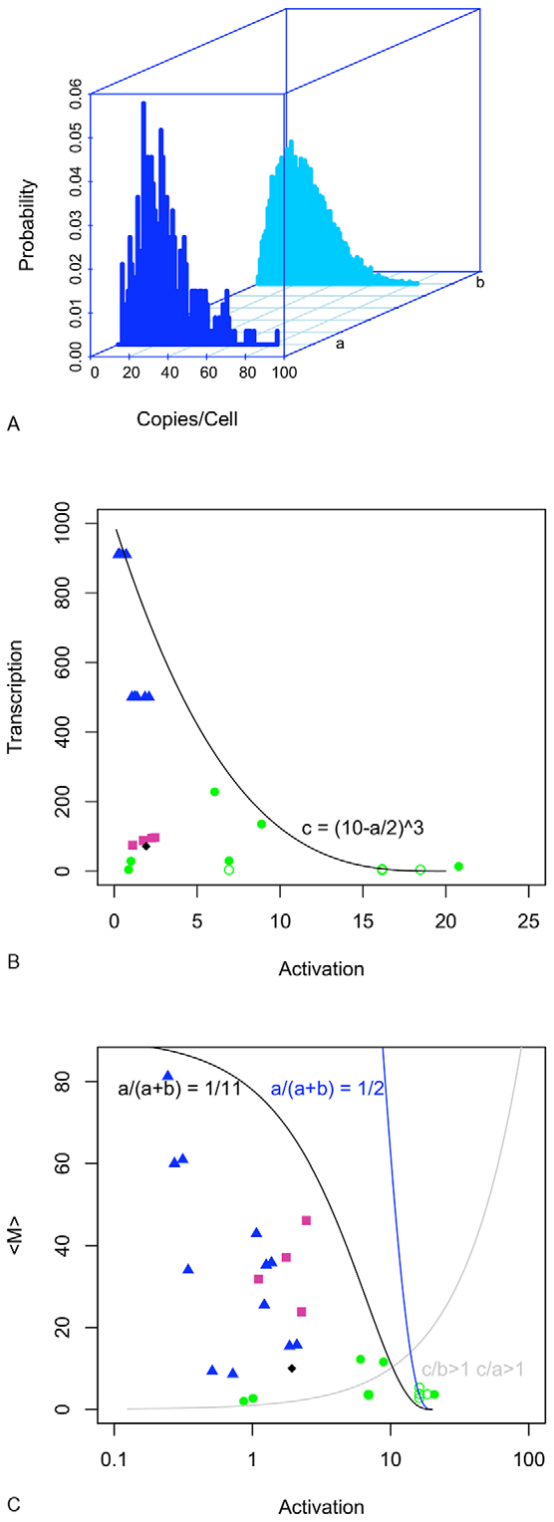
Modelling mRNA expression in single cells. A. Distribution of mRNA, a. observation and b. model. B. Scatterplot of 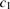 and 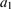 parameters from published models: blue triangles indicate mammalian data [4], green circles indicate yeast [7], as do purple squares (this paper), and the black diamond shows data from *E. coli* [2]. Closed symbols are plotted when 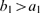, open symbols indicate 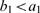. All parameters have been scaled such that 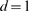 as in preceding figures. The line is an empirically-derived equation for the upper limit of 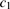. C. Scatterplot of 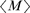 values and 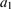 parameters from published models, symbols are defined as in B. Blue and black lines are the predicted upper constraint on 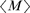 for alternative assumptions about the proportion of time in the on state. The grey line is 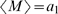.

We created a yeast strain that expresses a reporter RNA called Ribo1, and the number of mRNA per cell was counted using an in situ hybridization technique sufficiently sensitive to reliably detect single mRNA molecules (see Figure 7 and Materials and Methods). The reporter was under the control of a Tet-responsive promoter, contained the ACT1 intron and the 3′ processing signal of PGK1. Four cultures of yeast strains expressing Ribo1 were prepared and analyzed independently. In a representative sample, the mean number of mRNA/cell in steady-state was 23.8 copies/cell in a population of cells (327 cells, 2–82 copies/cell) The observed probability distribution is shown in Figure 8A, along with the predicted probability distribution under the parameterization listed in Table S4 (sample 4). As indicated in Table 1 , skewness and kurtosis are in good accord with observation when the variability of the data is accounted for by estimating the standard deviation by the bootstrap method. At these elevated mean mRNA levels, kurtosis becomes negative, i.e. the distribution is flatter than Gaussian. The fit of these characteristics to the data could be improved if skewness and kurtosis were optimized in model fitting.

**Table 1:** Skewness and kurtosis in the mRNA distribution in four sample populations, and estimates of these values from the corresponding on/off model (standard deviation).

Sample	No. Cells	Skewness (data)	Kurtosis (data)	Skewness (model)	Kurtosis (model)
1	152	0.2401 (0.1218)	−0.7203 (0.1641)	0.3499 (0.0174)	−0.5440 (0.0352)
2	378	0.3218 (0.0839)	−0.7119 (0.1467)	0.3558 (0.0211)	−0.7702 (0.0279)
3	315	0.0954 (0.0865)	−0.3972 (0.1339)	0.1924 (0.0138)	−0.5518 (0.0185)
4	327	1.0353 (0.1522)	1.1975 (0.5929)	0.7356 (0.0182)	0.3311 (0.0660)

Standard deviation of data is estimated from 1000 bootstrap samples.

To explore the possibility of constraints on model parameters, the Ribo1 data are included in the scatterplots in Figure 8 of *a* 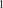 values from published models [2], [4], [7]. Values of synthesis 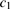 are plotted against 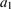 in 8B, and in 8C mean mRNA 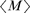 is plotted against activation rate. This analysis shows that, on the available evidence, there is a constraint on the synthesis rate that can be approximated by: 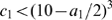. When this constraint is incorporated into equation 5, a constraint on 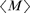 can be inferred when the ratio 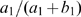 is considered. As this ratio increases, the possibility of high activation rates producing high levels of mRNA at low noise is reduced. The on/off model can be parameterized to give high copies/cell of mRNA with high activation rates (i.e. fast activation/inactivation cycles that reduce noise) but such a combination is not supported by the available data. Rather, high mean levels are modelled by low activation rates and are noisy. Low activation rates can also explain data where the mean mRNA is low. This evidence further supports the argument that regulation acts to increase time in the active state or to effectively increase the synthesis rate by regulating Pol II initiation or the efficiency of elongation, rather than to increase the frequency of activation [4]. These dominant factors are consistent with a processive model of elongation for activation rates below 2, and for high and low 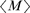. On the assumption that the noise implicit in the published model parameters are a true reflection of the data, we can rule out the highest values of *a* 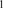 in the processive model as although they explain high mean mRNA, they underestimate the noise.

The ratio of synthesis to inactivation has been suggested as a way to define kinetic mechanisms [5], and to determine transcriptional bursting, i.e. 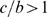 or 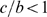. Some limitations of this approach have been noted [7]. The ratio of synthesis to activation might similarly be taken as defining a mode of gene expression: 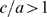 or 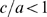. However, it is a straightforward consequence of equation 5 that for *a* 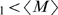 it must be the case that 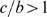 and 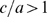. The boundary *a* 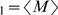 is shown in Figure 8C: Below this line only one of the inequalities in each pair can possibly hold, above it a second mode is possible. The ratio of activation to inactivation is another simple distinction that can be made, and this too is shown in Figure 8C by the use of open and closed symbols. It can be seen that 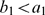 only for higher values of *a* 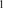. The ratios 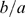, 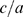 and 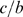 take a continuum of values that, broadly speaking, reduce as *a* 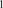 increases. None of these distinctions explain the absence of high *a* 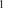 rates for high 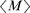, but we note that the assumption of coupled of elongation reveals that this would be, at the very least, a highly inefficient mode of transcription under such conditions, if not an impossibility.

## Discussion

The on/off model of mRNA transcription has been simulated over a wide range of feasible parameters. The distribution of mRNA/cell in steady-state has been summarised by the mean, second, third and fourth moments. Unlike the mean and variance, equations for skewness, kurtosis have not been derived analytically in the general case - and their application to the on/off model is presented here for the first time. Modifications to the on/off model that give a more detailed account of elongation are shown to influence all of these measures. Moreover, we have shown that skewness and kurtosis are of value when comparing models.

Each step in elongation, and the possibility of Pol II backtracking, is modelled in [19]. The count procedure in the models proposed here similarly captures the phenomenon of a repeated step-wise process, but does so in a way that allows the parameters of the on/off initiation model to be mapped to the new models, and surveyed. The representation of promoter state and elongation in a single model is also found in the model of the dynamics of Pol II engagement proposed by [26]. However, our model does not distinguish initiation from the later engagement process, nor assign them different rates in order to explore the assumptions we make in a uniform way across the parameter space.

The on/off model has been used successfully to explain single-cell mRNA data [1]–[7], without the modifications proposed here. Even where noise is observed to be low [7], it remains at least Poissonian and hence consistent with the model. While a deterministic elongation model reduces noise below this bound, processivity and coupling restore noise to the gene expression characteristic. Initiation, pausing and backtracking in elongation further reduce its predictability, and accounting for them in a processive model will add additional noise to the process of transcription. The marginal impact that different stochastic processes have on the overall size of fluctuations in a population is noted in [32], where protein noise is shown to be only weakly dependent on burst size and waiting time statistics, and on the number of steps in the degradation pathway. Through this course-graining effect [32], the phenomenological on/off model may indeed accurately summarise the molecular mechanisms that are being uncovered and modelled. One critical step will be to accurately determine the activation and synthesis rates experimentally, e.g. as in [2], as without this data they must be found by optimization. As illustrated in this paper, the distributions that they are estimated from are essentially invariant in certain regions of parameter space. A further issue arises in model comparison, where the saturated elongation process necessary to maintain a high mRNA expression level may obscure any characteristics that result from molecular-level elongation mechanisms: Even with full knowledge of the important parameters, the predictions are likely to be dominated by the activation/inactivation cycle rather than by the more subtle features of the synthesis process at lower levels of processing. From the modelling perspective, genes expressed at low levels are the more promising candidates for testing alternative theories, but clearly this poses challenges experimentally.

## Materials and Methods

### Stochastic Simulation

All simulations were performed using the Gillespie algorithm implemented in Dizzy (http://magnet.systemsbiology.net/software/Dizzy/). This software was modified to store the histograms of the distributions for further analysis. 10,000 simulations were run for each set of parameters surveyed, and this procedure was repeated 10 times to obtain means and variances. The EDDIE compute cluster was used to run the simulations, which, for the more complex models, took 7–10 hours for each parameter survey, each of which was repeated 10 times for the four models.

It is necessary to show that the simulations have stabilised, and this was achieved by measuring the divergence between the distribution for the end time and that of an earlier time using the Kullback-Leibler (KL) measure:
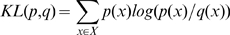
(8)


The KL divergence is calculated from the probability that the data falls into a bin (*x*) as defined in the two distributions being compared, *p* and *q*. The divergence between the probability distribution of mRNA at 50s and at 33s (of simulated time) is plotted in Figure 3 in order to demonstrate that the simulation has reached a stationary state. KL divergence is also useful for determining how good a fit the best-fitting Poisson, Gaussian, gamma or negative binomial distribution is to the simulated distribution. The best-fit distribution parameters are calculated from the simulation data using maximum likelihood, but this calculation alone does not return a goodness of fit, and so the distance must be calculated separately. The SSJ Java library (http://www.iro.umontreal.ca/simardr/ssj/indexe.html) was used to obtain the distribution parameters. The KL divergence is a measure of relative entropy [33], decreasing as the distributions become more similar. It is asymmetric, and weights the terms in the summation by *p(x)*. The measure does not allow the hypothesis that the distributions are the same to be tested at a specified P value. For this, we chose the one-sided Chi-square test at the P value 0.05

### Skewness and Kurtosis

The skewness and kurtosis of a set of 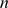 samples 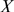 with mean 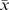 and standard deviation 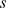 is defined as follows:
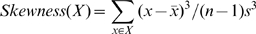
(9)

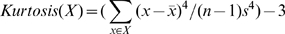
(10)


The subtraction of 3 from the sum in equation 10 makes the value of kurtosis for a Gaussian distribution equal to 0.

Skewness is a measure of asymmetry and is 0 for a symmetric distribution such as the Gaussian distribution. Negative values indicate a longer tail to the left of the peak, while positive values indicate that the right tail is longer. Kurtosis measures how peaked the distribution is, relative to the Gaussian distribution (the subtraction of 3 from the sum in equation 10 makes the value of kurtosis for a Gaussian distribution equal to 0). Positive values for kurtosis indicate that a distribution is more peaked than the Gaussian. Negative values indicate a distribution that is flatter than the Gaussian distribution.

### Image-Based Measurement of mRNA in Yeast

The Ribo1 reporter was integrated as a single copy at the his3 locus. Yeast strains were grown in YNB and fixed in mid-log phase. The sequence of the probes and the in situ hybridization protocol used in this study are posted on the Ribosys project website http://www.ribosys.org. We used a semi-automated procedure to detect and automatically count single molecules of the various reporter mRNAs. This procedure relies on the fact that single mRNA molecules are visible as bright, individual spots. 3D stacks of control and strains expressing the various reporters were taken with a CCD camera (CoolSnap HQ, Roper Scientific), on an upright microscope equipped for fluorescent imaging (DMRA, Leica microsystems), with a 100× objective, NA 1.4. Image analysis was performed with the Spot function of Imaris 3.0 (Bitplane). A Gaussian filter 0.2 micrometer wide was first used to remove variation in local intensities smaller than spots of single mRNAs, see Figure 7B. Then, all local maxima were identified in the 3D image, and the maxima corresponding to background were removed by setting a minimal threshold for the local variation in intensity. This threshold varied from experiment to experiment, and it was chosen as the minimal value that eliminated all spots from the control yeast strain (see Figure 7B). To evaluate the efficiency of detection, we plotted histograms of the number of spots detected as a function of local spot intensity (see Figure 7C). This allowed us to estimate that we detected more than 85% of the mRNA molecules, with no background contamination. In each case, the distribution were derived from 110 to 358 cells.

## Supporting Information

Figure S1 Skewness and kurtosis. For the on/off model: A. <M> = 1, and B. for <M> = 10. For the on/off-PE model: C. <M> = 1, and D. for <M> = 10. For the on/off-CE model: E. <M> = 1, and F. for <M> = 10. Error bars show the standard deviation for 10 repetitions.
(0.13 MB TIF)

Figure S2 Scatterplots of skewness and kurtosis for the on/off-PE model taking the on/off model as a reference. <M> = 10 in both cases: A. skewness; B. kurtosis. Points are average values from 10 repetitions. Solid grey line indicates y = x.
(0.96 MB TIF)

Table S1 Estimates of skewness, kurtosis and Kullback-Leibler divergence from sampling a Poisson distribution (λ = 9). Mean and variance are obtained from 1000 repetitions. This table presents the results of a computational experiment where between 100 and 100,000 samples are drawn from a Poisson distribution and the skewness, kurtosis and KL divergence are calculated. The value of λ is 9 in the generating distribution, and therefore the theoretical value of skewness is 1/3, kurtosis is 1/9 , and the theoretical mean is 9 in all samples. The table gives the mean and variance for these measures based on 1000 repetitions, and lists the error. For skewness and kurtosis, the error is the magnitude of the difference between the mean and the theoretical value, and for KL divergence the error is the factor by which the divergence increases in comparison with the divergence for the larger sample size (i.e., that given in the row above). This exploration indicates that skewness can be calculated reasonably accurately for sample sizes of 100 and above, while kurtosis may require a greater number, possibly as many as 1000 samples. For KL divergence, the difference between a sampled distribution and the theoretical distribution (which is known in this case) increases by a factor of approximately 8 for a ten-fold reduction in the number of samples. Therefore, skewness and kurtosis appear to be robust measures with respect to sample size, while KL divergence suffers from the bin-to-bin variability that results from lower sample sizes.
(0.02 MB PDF)

Table S2 Values of a_s_ scaled for d = 1s, 1min, 10min, 60min and 240min and expressed as mean times (s or h:m:s) for the transition.
(0.04 MB PDF)

Table S3 Values of c_s_ scaled for d = 1s, 1min, 10min, 60min and 240min (*values>0.5 are not realistic).
(0.03 MB PDF)

Table S4 Parameters for the on/off model for four sample populations of Ribo1.
(0.03 MB PDF)

Appendix S1 On/off models in Dizzy syntax.
(0.03 MB PDF)
