# Supplementary figures and images for: Processivity and Coupling in Messenger RNA Transcription
Source: PLoS One. 2010 Jan 28;5(1):e8845. doi: 10.1371/journal.pone.0008845 (PMC2812496; doi:10.1371/journal.pone.0008845)

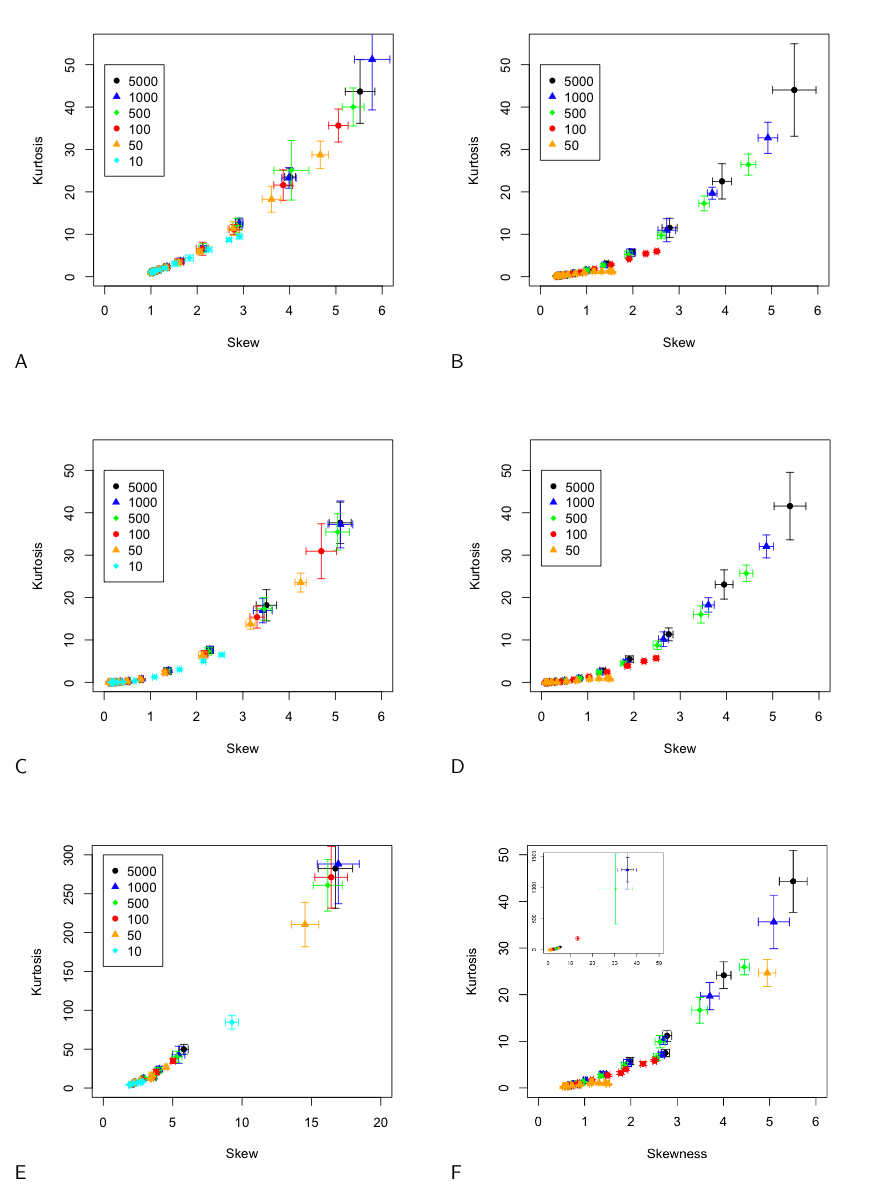

Supplement: Figure S1 — Skewness and kurtosis. For the on/off model: A. = 1, and B. for = 10. For the on/off-PE model: C. = 1, and D. for = 10. For the on/off-CE model: E. = 1, and F. for = 10. Error bars show the standard deviation for 10 repetitions. (0.13 MB TIF) [file pone.0008845.s001.tif]

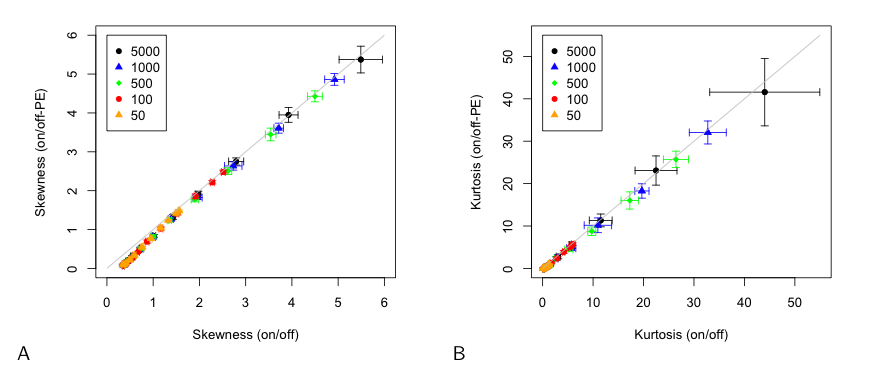

Supplement: Figure S2 — Scatterplots of skewness and kurtosis for the on/off-PE model taking the on/off model as a reference. = 10 in both cases: A. skewness; B. kurtosis. Points are average values from 10 repetitions. Solid grey line indicates y = x. (0.96 MB TIF) [file pone.0008845.s002.tif]
