# Supplementary material for: Processivity and Coupling in Messenger RNA Transcription
Source: PLoS One. 2010 Jan 28;5(1):e8845. doi: 10.1371/journal.pone.0008845 (PMC2812496; doi:10.1371/journal.pone.0008845)
Supplement: Table S1 — Estimates of skewness, kurtosis and Kullback-Leibler divergence from sampling a Poisson distribution (λ = 9). Mean and variance are obtained from 1000 repetitions. This table presents the results of a computational experiment where between 100 and 100,000 samples are drawn from a Poisson distribution and the skewness, kurtosis and KL divergence are calculated. The value of λ is 9 in the generating distribution, and therefore the theoretical value of skewness is 1/3, kurtosis is 1/9 , and the theoretical mean is 9 in all samples. The table gives the mean and variance for these measures based on 1000 repetitions, and lists the error. For skewness and kurtosis, the error is the magnitude of the difference between the mean and the theoretical value, and for KL divergence the error is the factor by which the divergence increases in comparison with the divergence for the larger sample size (i.e., that given in the row above). This exploration indicates that skewness can be calculated reasonably accurately for sample sizes of 100 and above, while kurtosis may require a greater number, possibly as many as 1000 samples. For KL divergence, the difference between a sampled distribution and the theoretical distribution (which is known in this case) increases by a factor of approximately 8 for a ten-fold reduction in the number of samples. Therefore, skewness and kurtosis appear to be robust measures with respect to sample size, while KL divergence suffers from the bin-to-bin variability that results from lower sample sizes. (0.02 MB PDF) [file pone.0008845.s003.pdf]

No. Samples	Skewness		Kurtosis		KL divergence	
	Mean (variance)	Error	Mean (variance)	Error	Mean (variance)	Error
100,000	0.3338 (6.22E-5)	0.0005	0.1118 (4.82E-4)	0.0007	1.706E-4 (2.36E-9)	-
10,000	0.3328 (6.50E-4)	0.0006	0.1104 (4.87E-3)	0.0007	0.0016 (2.57E-7)	*9.1
1000	0.3306 (6.22E-3)	0.0027	0.09996 (4.84E-2)	0.0111	0.0133 (2.21E-5)	*8.6
500	0.3241 (1.37E-2)	0.0093	0.0904 (9.8E-2)	0.0207	0.0252 (8.58E-5)	*1.9
100	0.3161 (6.25E-2)	0.0173	0.022 (0.347)	0.0891	0.1005 (1.81E-3)	*4.0
