# Supplementary material for: Processivity and Coupling in Messenger RNA Transcription
Source: PLoS One. 2010 Jan 28;5(1):e8845. doi: 10.1371/journal.pone.0008845 (PMC2812496; doi:10.1371/journal.pone.0008845)
Supplement: Table S2 — Values of as scaled for d = 1s, 1min, 10min, 60min and 240min and expressed as mean times (s or h:m:s) for the transition. (0.04 MB PDF) [file pone.0008845.s004.pdf]

| $a_s$ | $a_1$ | | | | | | | | | | |
|---|---|---|---|---|---|---|---|---|---|---|---|
| $d_s$ | 1/8 | 1/4 | 1/2 | 1 | 2 | 4 | 8 | 16 | 32 | 64 | 128 |
| 1 | 8 | 4 | 2 | 1 | 1/2 | 1/4 | 1/8 | 1/16 | 1/32 | 1/64 | 1/128 |
| 1/60 | 480 | 240 | 120 | 60 | 30 | 15 | 7.5 | 3.75 | 1.875 | 0.9375 | 0.47 |
| 1/600 | 1:20:0 | 2400 | 1200 | 600 | 300 | 150 | 75 | 37.5 | 18.75 | 9.38 | 4.69 |
| 1/3600 | 8:0:0 | 4:0:0 | 2:0:0 | 1:0:0 | 1800 | 900 | 450 | 225 | 112.5 | 56.25 | 28.1 |
| 1/14400 | 32:0:0 | 16:0:0 | 8:0:0 | 4:0:0 | 2:0:0 | 1:0:0 | 1800 | 900 | 450 | 225 | 112.5 |
