# Supplementary material for: Processivity and Coupling in Messenger RNA Transcription
Source: PLoS One. 2010 Jan 28;5(1):e8845. doi: 10.1371/journal.pone.0008845 (PMC2812496; doi:10.1371/journal.pone.0008845)
Supplement: Table S3 — Values of cs scaled for d = 1s, 1min, 10min, 60min and 240min (*values>0.5 are not realistic). (0.03 MB PDF) [file pone.0008845.s005.pdf]

c_s	c_1					
d_s	5000	1000	500	100	50	10
1	5000*	1000*	500*	100*	50*	10*
1/60	83.3*	16.7*	8.3*	1.67*	0.83*	0.167
1/600	8.33*	1.67*	0.83*	0.167	0.083	0.0167
1/3600	1.39*	0.28	0.14	0.028	0.014	0.0028
1/14400	0.347	0.069	0.035	0.0069	0.0035	0.00069
