# Supplementary material for: Processivity and Coupling in Messenger RNA Transcription
Source: PLoS One. 2010 Jan 28;5(1):e8845. doi: 10.1371/journal.pone.0008845 (PMC2812496; doi:10.1371/journal.pone.0008845)
Supplement: Table S4 — Parameters for the on/off model for four sample populations of Ribo1. (0.03 MB PDF) [file pone.0008845.s006.pdf]

Sample	a_1	b_1	c_1	d_1
1	1.75762	2.41654	88.12865	1
2	1.10872	1.49679	74.78043	1
3	2.45573	2.70479	96.95541	1
4	2.26735	6.81516	95.42599	1
