# Supplementary material for: Processivity and Coupling in Messenger RNA Transcription
Source: PLoS One. 2010 Jan 28;5(1):e8845. doi: 10.1371/journal.pone.0008845 (PMC2812496; doi:10.1371/journal.pone.0008845)
Supplement: Appendix S1 — On/off models in Dizzy syntax. (0.03 MB PDF) [file pone.0008845.s007.pdf]

## Models in Dizzy syntax

```
on/off model
----------
 a = 1;
 b = 99;
 c = 500;
 d = 1;
 ACTIVE=1;
 INACTIVE=0;
 M=0;
 ACTIVE -> INACTIVE, b;
 INACTIVE -> ACTIVE, a;
 ACTIVE ->ACTIVE + M, [min(1,ACTIVE)*c];
 M ->, d;

on/off-DE model
--------------
 a = 1;
 b = 99;
 c = 500;
 d = 1;
 NOCOUNTS = 0;
 counttotal = 100;
 ACTIVE=1;
 INACTIVE=0;
 M=0;
 COUNT = 0;
 ISCOUNTING = 0;
 ACTIVE -> INACTIVE,b;
 INACTIVE -> ACTIVE,a;
 ACTIVE -> ACTIVE + COUNT + ISCOUNTING,[min(1,ACTIVE)*min(1,1-ISCOUNTING)*counttotal*c];
 COUNT + ISCOUNTING -> COUNT + COUNT + ISCOUNTING, [min(1,ACTIVE)*min(1,ISCOUNTING)*counttotal*c];
 COUNT + ISCOUNTING -> M + COUNT + COUNT + NOCOUNTS,
    [min(1,ACTIVE)*min(1,ISCOUNTING)*min(1,max(0,(COUNT+2-(NOCOUNTS*counttotal+counttotal))))*counttotal*c];
 M ->,d;
```

```
on/off-PE model
--------------
 a = 1;
 b = 99;
 c = 500;
 d = 1;
 NOCOUNTS = 0;
 counttotal = 100;
 ACTIVE=1;
 INACTIVE=0;
 M=0;
 COUNT = 0;
 ISCOUNTING = 0;
 ACTIVE -> INACTIVE,b;
 INACTIVE -> ACTIVE,a;
 ACTIVE -> ACTIVE + COUNT + ISCOUNTING,[min(1,ACTIVE)*min(1,1-ISCOUNTING)*counttotal*c];
 COUNT + ISCOUNTING -> COUNT + COUNT + ISCOUNTING, [min(1,ISCOUNTING)*counttotal*c];
 COUNT + ISCOUNTING -> M + COUNT + COUNT + NOCOUNTS,
    [min(1,ISCOUNTING)*min(1,max(0,(COUNT+2-(NOCOUNTS*counttotal+counttotal))))*counttotal*c];
 M ->,d;

on/off-CE model
--------------
 a=1;
 b=99;
 c = 500;
 d = 1;
 NOCOUNTS = 0;
 counttotal = 100;
 ACTIVE=1;
 INACTIVE=0;
 M=0;
 COUNT = 0;
 ISCOUNTING = 0;
 ACTIVE -> INACTIVE,b;
 INACTIVE -> ACTIVE,a;
 ACTIVE -> ACTIVE + COUNT + ISCOUNTING,[min(1,ACTIVE)*min(1,1-ISCOUNTING)*counttotal*c];
 COUNT + ISCOUNTING -> COUNT + COUNT + ISCOUNTING, [min(1,ACTIVE)*min(1,ISCOUNTING)*counttotal*c];
 COUNT -> , [min(1,INACTIVE)*min(1,max(0,COUNT-NOCOUNTS*counttotal))*counttotal*c];
 COUNT + ISCOUNTING -> M + COUNT + COUNT + NOCOUNTS,
    [min(1,ACTIVE)*min(1,ISCOUNTING)*min(1,max(0,(COUNT+2-(NOCOUNTS*counttotal+counttotal))))*counttotal*c];
 M ->,d;
```
